# Lymphocyte Activation Dynamics Is Shaped by Hereditary Components at Chromosome Region 17q12-q21

**DOI:** 10.1371/journal.pone.0166414

**Published:** 2016-11-11

**Authors:** Amado Carreras-Sureda, Fanny Rubio-Moscardo, Alex Olvera, Jordi Argilaguet, Kerstin Kiefer, Beatriz Mothe, Andreas Meyerhans, Christian Brander, Rubén Vicente

**Affiliations:** 1 Laboratory of Molecular Physiology, Department of Experimental and Health Sciences, Universitat Pompeu Fabra, Barcelona, Spain; 2 Biomedical Neuroscience Institute, Faculty of Medicine, University of Chile, Santiago, Chile; 3 Program of Cellular and Molecular Biology, Center for Molecular Studies of the Cell, Institute of Biomedical Sciences, University of Chile, Santiago, Chile; 4 AIDS Research Institute, IrsiCaixa—HIVACAT, Hospital Germans Trias i Pujol, Badalona, Spain; 5 Infection Biology Group, Department of Experimental and Health Sciences, Universitat Pompeu Fabra, Barcelona, Spain; 6 Universitat de Vic-Universitat Central de Catalunya (UVic-UCC), Vic, Spain; 7 Institució Catalana de Recerca i Estudis Avançats (ICREA), Barcelona, Spain; Pohang University of Science and Technology, REPUBLIC OF KOREA

## Abstract

Single nucleotide polymorphisms (SNPs) located in the chromosome region 17q12-q21 are risk factors for asthma. Particularly, there are *cis*-regulatory haplotypes within this region that regulate differentially the expression levels of *ORMDL3*, *GSDMB* and *ZPBP2* genes. Remarkably, *ORMDL3* has been shown to modulate lymphocyte activation parameters in a heterologous expression system. In this context, it has been shown that Th2 and Th17 cytokine production is affected by SNPs in this region. Therefore, we aim to assess the impact of hereditary components within region 17q12-q21 on the activation profile of human T lymphocytes, focusing on the haplotype formed by allelic variants of SNPs rs7216389 and rs12936231. We measured calcium influx and activation markers, as well as the proliferation rate upon T cell activation. Haplotype-dependent differences in mRNA expression levels of IL-2 and INF-γ were observed at early times after activation. In addition, the allelic variants of these SNPs impacted on the extent of calcium influx in resting lymphocytes and altered proliferation rates in a dose dependent manner. As a result, the asthma risk haplotype carriers showed a lower threshold of saturation during activation. Finally, we confirmed differences in activation marker expression by flow cytometry using phytohemagglutinin, a strong polyclonal stimulus. Altogether, our data suggest that the genetic component of pro-inflammatory pathologies present in this chromosome region could be explained by different T lymphocyte activation dynamics depending on individual allelic heredity.

## Introduction

The genetic component behind the susceptibility of some individuals to certain diseases is based on polymorphisms within the human genome that can modify the function and/or the expression levels of one or more genes. Genome Wide Association Studies (GWAS) search for unbalanced distributions of allelic frequencies of Single Nucleotide Polymorphisms (SNPs) that point out novel genes associated to complex diseases. These SNPs often do not locate within coding regions, but map to *cis* regulatory elements that affect expression levels of genes surrounding them. This is the case for Orosomucoid-like 3 (*ORMDL3*), a member of the orosomucoid-like protein family (ORMDL) associated to childhood asthma [1]. The SNP rs7216389, claimed to be a risk factor for asthma, is present in the intron of the neighboring gene Gasdermin B (*GSDMB)* and has been shown to modulate expression of both genes. Various SNPs in the same chromosome region 17q12-q21 form a *cis* regulatory haplotype in linkage disequilibrium that determines, by altering nucleosome enrichment and methylation, the expression of adjacent genes such as IKAROS Family Zinc Finger 3 (*IKZF3)*, Zona Pellucida Binding Protein 2 (*ZPBP2)*, *GSDMB* and *ORMDL3*[2,3].

The chromosome region 17q12-q21 is a hereditary component for several inflammatory diseases besides asthma. SNPs located here are associated to Crohn’s disease, ulcerative colitis, ankylosing spondylitis, type I diabetes or rheumatoid arthritis [4–8]. Moreover, white blood cells counts [9], IL-17, IL-4 and IL-13 production [10,11] are also associated with SNPs present in this region. Thus, chromosome 17q12-q21 carries important genetic regulatory elements of the immune system. The function of two of the genes located on chromosome 17q12-q21, *ORMDL3* and *IKZF3*, is related to immune system physiology. For example, *ORMDL3* expression levels have been shown to influence T cell activation by altering calcium homeostasis and the Store Operated Calcium Entry (SOCE) pathway in a Jurkat T cell model [12,13] as well as to alter eosinophil function [14]. *IKZF3* is a transcription factor with an important role in lymphocyte differentiation and apoptosis [15–17]. For the other 2 genes, *ZPBP2* and *GSDMB*, no data are available to support or disprove a link to immune cell functioning. Interestingly, Gasdermin *D* (*GSDMD*), a different member of the gasdermin family, is an important component of inflammasomes and participates in pyroptosis. While *GSDMB* shares the same structural domains, it is still unknown whether it is also involved in the same pathway and therefore plays a role in immune function [18,19].

In the present work we focused on T lymphocyte activation, a key process for the correct tuning of the immune response. T cell activation is dependent on two main signaling cascades. The first one is the T Cell Receptor (TCR) signaling pathway that triggers the early activation program and allows a linear correlation between antigen dose and activation markers like IL-2 production and IL-2 receptor (CD25) expression. The second signaling pathway comes from the autocrine and paracrine regulation generated by IL-2, which promotes T cell clone expansion[20,21]. The IL-2 cascade has positive and negative feedback loops that allow scaling the clonal density and immune response to a wide range of antigenic loads [22]. Alterations in these two signaling pathways can modify the T cell response altering parameters like threshold of activation or clonal expansion balance that can end up in dysfunctional reactivity and autoimmune processes [23].

Taking into consideration the chromosome 17q12-q21-associated phenotypes with immune-related pathologies, we aimed to explore the genetic contribution of this region to T cell activation. Accordingly, we isolated peripheral lymphocytes from donors with allelic differences in SNPs rs7216389 and rs12936231; and studied nearby gene expression during activation. Moreover, by monitoring several markers we could measure the kinetics of activation and analyzed T cell proliferation. Our work shows that the allelic variability within this chromosome region correlates with the kinetics and the degree of T lymphocyte activation.

## Results

### Gene expression in region 17q12-q21 is modulated by hereditary components

**Fig 1:**
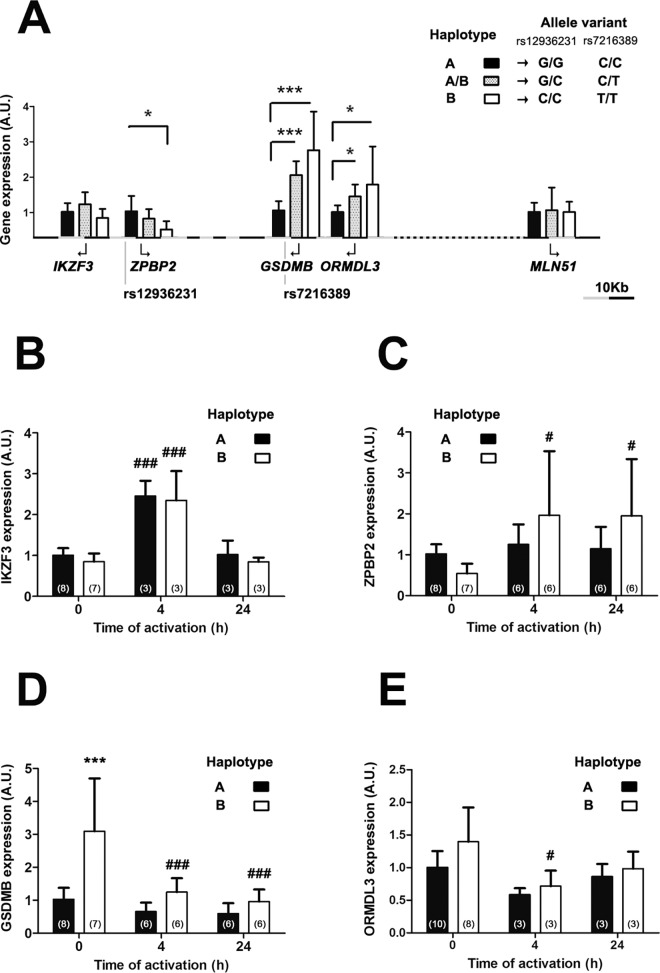
Expression levels of genes in chromosome region 17q12-q21. Expression levels of *IKZ3F*, *ZPBP2*, *GSDMB*, *ORMDL3* and *MLN51* in resting human T cells are normalized to the housekeeping gene *HPRT* and classified according to the haplotypes defined by SNPs: rs7216389 and rs12936231. X axes represent the distance (grey-black subdivision corresponds to 10Kb) in the region between SNPs and the first exon of each gene. The number of individuals of haplotype A and haplotype B carriers was the same as indicated at time 0h in panels B-E. n = 3 in haplotype AB carriers. Data presented as mean ± SD. Statistics using *t* test statistic analysis; *p<0.05, ***p<0.001. (**A**). Gene expression levels of *IKZ3F* (B), *ZPBP2* (C), *GSDMB* (D) and *ORMDL3* (E) in CD3/CD28-activated T cells at the indicated time points. Expression data during activation are normalized to the housekeeping gene *MNL51* (**B-E**). Arbitrary Units (A.U.) Number of individuals indicated in brackets. Data presented as mean ± SD. Statistics using Bonferroni ANOVA analysis; ***p<0.001 between haplotypes at the same time point. #p<0.05, ###p<0.001 compared to time 0h.

It has been previously reported that SNPs in the chromosome region 17q12-q21 form a *cis* regulatory haplotype that changes the expression levels of genes within this region [1,2]. In this work, in order to experimentally define this haplotype in linkage disequilibrium we chose the SNPs rs7216389, the first asthma associated SNP described in this region [1], and rs12936231, an evolutionary conserved SNP whose allelic variants have been postulated to contribute to alternative conformations of the chromosome region 17q12-q21 [2]. The strong linkage existing between these different SNPs is highlighted by the fact that in our working sample the total of C and G carriers in SNP rs12936231 were T and C carriers in SNP rs7216389 respectively [2]. We then evaluated the expression of different genes of this region in human lymphocytes under resting and activation conditions depending on haplotypes defined by the allele combination of these two SNPs: haplotype A were homozygous G (rs12936231) and C (rs7216389) carriers, and haplotype B were homozygous C (rs12936231) and T (rs7216389) carriers. Haplotype B contains the allelic combination that causes risk for asthma [1] (Fig 1A). We also included a heterozygous population carrying both haplotypes that we called haplotype A/B carriers. The genes studied included *IKZF3*, *ZPBP2*, *GSDMB*, *ORMDL*3 and *MLN51*. The comparative analysis by RT-PCR of peripheral T cells showed an increased transcription of *ORMDL3* and *GSDMB*, and decreased *ZPBP2* transcription in resting lymphocytes of haplotype B carriers *vs* haplotype A carriers. *IKZF3* and *MLN51* transcription was not affected by the different allelic combinations (Fig 1A). Haplotype A/B carriers showed an intermediate expression pattern significantly different from haplotype A carriers for *ORMDL3* and *GSDMB* expression levels.

We further studied gene expression analysis during activation using anti-CD3/CD28 antibodies. *MLN51* is an excellent housekeeping gene during lymphocyte activation [24–26] and its expression is not dependent on the studied haplotypes at any time point of activation (S1 Fig). We have used it to normalize gene expression levels under activation conditions. Ours results showed that the expression differences between haplotypes of the different genes at resting conditions were lost upon induction of lymphocyte activation at early (4h) and late (24h) time points (Fig 1B–1E). Furthermore, *GSDMB* in haplotype B carriers showed a marked repression of transcription after T cell activation. On the contrary, *IKZF3* showed a clear early induction during activation (Fig 1B–1E).

### Store-Operated Calcium Entry varies between populations

**Fig 2:**
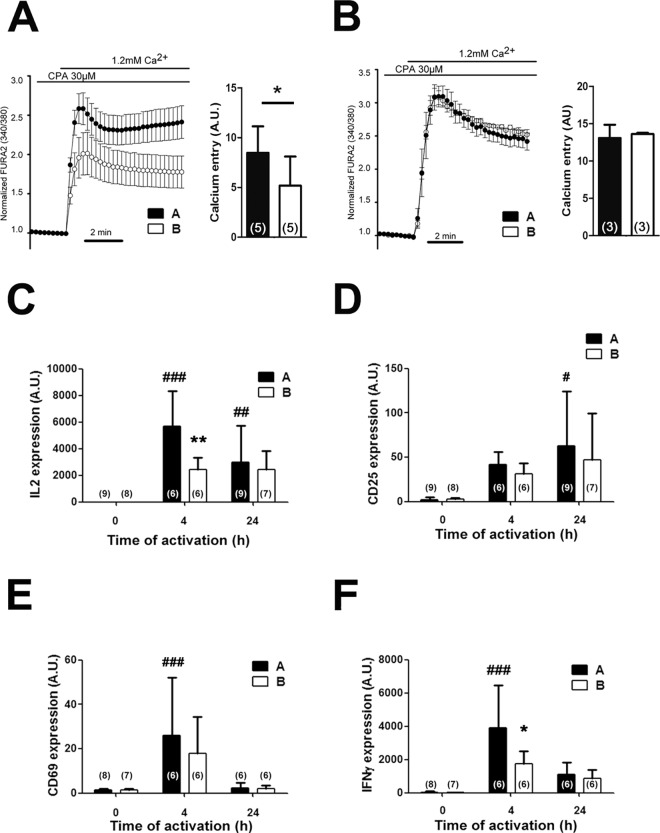
Haplotype-dependent kinetics of T cell activation. **A**. Store-operated calcium entry (SOCE) after store depletion with CPA of resting cells or (**B**) in T cells after 24 hours of CD3/CD28 treatment. Bars show calcium entry, analyzing the area under the curve in Arbitrary Units (A.U.) Data presented as mean ± SD. Statistics using *t* test statistic analysis; *p<0.05. **C-F**. Longitudinal gene expression analysis of human T cells stimulated with CD3/CD28 at resting, 4h and 24 hours normalized to the housekeeping gene *MLN51* expression. **C**. IL-2 RNA expression. **D**. CD69 RNA expression. **E**. CD25 RNA expression. **F**. IFNγ RNA expression. Number of individuals indicated in brackets. Data presented as mean ± SD. Statistics using Bonferroni ANOVA analysis; *p<0.05, **p<0.01 between haplotypes at the same time point. #p<0.05, ##p<0.01, ###p<0.001 compared to time 0h.

Our laboratory has previously shown that expression levels of ORMDL3 are inversely correlated with the SOCE pathway [13], a key signaling event in the activation of T cells. We thus evaluated the impact of haplotype A *vs* B on SOCE triggered by store depletion in resting and activated lymphocytes from healthy human volunteers. Our calcium analyses showed that in resting lymphocytes, SOCE is reduced in homozygous carriers of the asthma risk haplotype B compared to haplotype A carriers (Fig 2A). This difference, however, was lost after activation for 24h with anti-CD3/CD28 antibodies (Fig 2B).

### The chromosome region 17q12-q21 defines a threshold of saturation under TCR stimulation

**Fig 3:**
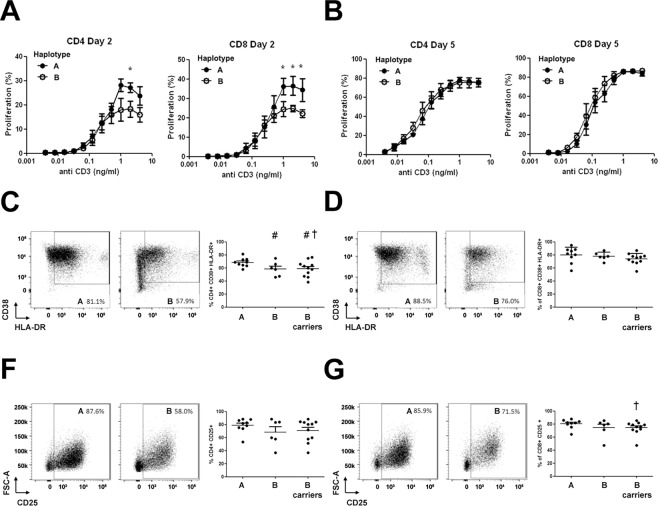
Proliferation and activation differences between the haplotype variants. Percentages of proliferating CD4+ and CD8+ lymphocytes at days 2 (**A**) and 5 (**B**) after stimulation of CFSE-labeled PBMCs with decreasing doses of anti-CD3 antibody. Experiment performed with 4 individuals for each haplotype. *t* test statistic analysis; *p<0.05. **C-G**, Activation analysis by flow cytometry after 72h of culture with and without PHA stimulation measuring percentage of CD38+ HLA-DR+ (C-D) or CD25+ (F-G) cells in CD4+ (C-F) and CD8+ (D-G) T lymphocytes from individuals with A and B haplotype. Included is also a group of “B carriers” which comprises all individuals homozygous and heterozygous for the B haplotype. Statistics using *t* test with (†p<0.05) and without (#p<0.05) Welch correction.

Given that a reduction in calcium entry might shape the activation kinetics of T lymphocytes, we evaluated different markers of T cell activation from volunteers with allelic differences. We measured the mRNA expression of IL-2, CD25, CD69 and IFN- γ by RT-PCR at different time points after T cell activation with anti-CD3/CD28 stimulation. Our results showed a significant reduction in IL-2 and in IFN-γ expression in haplotype B carriers, compared to haplotype A carriers at 4h (Fig 2C and 2F). These differences were no longer detected at 24h post-stimulation. CD25 and CD69 showed a similar trend during activation but the difference was not significant between haplotypes (Fig 2D and 2E). These results are in agreement with the lower calcium entry into resting lymphocytes and might indicate that lymphocyte from haplotype B carriers have an increased activation threshold. In order to study this possibility, we performed dose-dependent proliferation studies of peripheral T lymphocytes by flow cytometry (Fig 3). Lymphocytes from haplotype B carriers presented a lower proliferation rate at day 2 under higher concentrations of anti CD3 stimulation in CD4+, and more evident, in CD8+ lymphocytes (Fig 3A). However, there were no differences either at day 2 or 5 of proliferation at low anti-CD3 concentrations (Fig 3B).

We further studied by flow cytometry the lymphocyte activation using peripheral blood mononuclear cells (PBMC) activated with the lectin phytohaemagglutinin (PHA), another strong stimulus that signals like anti CD3 antibodies in a calcium dependent manner (Fig 3C–3G). We observed a significant reduction of CD38+ HLA-DR+ cells within CD4+ lymphocytes (Fig 3C) and a reduced number of CD25+ cells within CD8+ lymphocytes for the haplotype B variant (Fig 3F). Combining heterozygous AB and homozygous B carriers (named as B carriers) allowed us to strengthen the analysis observing in all cases the trend to have lower activation degree in B carriers compared to those with haplotype A.

## Discussion

In the present work, we have addressed the potential role of T lymphocyte activation in the described association between SNPs in chromosome region 17q12 and asthma. First, we have measured the expression of genes surrounding SNP rs12936231 and rs7216389 in order to evaluate their influence on the T lymphocyte activation pathway. The allelic analysis of both SNPs in our population confirmed that they are in linkage disequilibrium forming haplotypes previously described [2,3]. Our analyses further confirmed that the regulation of *ORMDL3*, *GSDMB*, *ZPBP2* and *IKZF3* expression in non-stimulated cells is haplotype dependent [2], thus validating our sampling cohort. Remarkably, we found a loss of expression regulation depending on the haplotype during the activation process suggesting that other elements in the promoter, induced by the activation cascade, might acquire dominancy independent of the SNPs allelic variants. Similar results have been previously observed by others using PHA as an activator [10,11]. However, it has been also described that lymphocyte activation using Derp 1, an allergen of *D*. *pteronyssinus*, produces a differential gene expression within the region depending on the haplotype. All these data suggest a complex transcriptional control scenario of the 17q12-q21region linked to the stimulus applied and the pathways involved [11].

Regarding the activation process, our results showed that the activation profile of T cells varied in a dose- and time-dependent manner attending to the haplotype present in the 17q12-q21 region. B haplotype carriers, considered carriers of the asthma associated haplotype [1], revealed a reduced expression of activation markers and a lower proliferation under strong stimulation. A study based on computational analysis of the activation phases in lymphocytes allows to better understand our results [21]. Thus, the T cell activation program fully relies in the first hours on the T cell receptor (TCR) signaling pathway, producing a rise in cytosolic calcium that triggers the translocation of transcription factors such as NF-AT in order to express essential players like IL-2 and its receptor CD25[21]. As a consequence, in the first 24 h a population of cells expressing low amounts of CD25 appears (CD25L cells). From 19 h on, a second pathway of activation occurs mainly driven by the IL-2 cascade that through a positive feedback loop further promotes the expression of CD25, appearing as proliferative and high expressing CD25 cells (CD25H cells), but represses in a negative feedback loop the expression of IL-2 [20]. Interestingly, the dose dependent proliferation is established in the early TCR dependent phase, determining the amount of CD25L positive cells at 24 h that will be translated proportionally in the proliferating CD25H positive cells at later time points [21]. Our results fit in this scenario because we found major differences in the TCR driven early phase such as calcium signaling, IL-2 and IFN-γ production that later determine the proliferation rate. The questions that arise from our results are related to the concepts of threshold of activation and of saturation, both of them relying in the transduction process triggered upon TCR activation [21]. It has been shown that defects in co-stimulatory elements of the TCR complex like CD28 can modify the threshold of activation [23]. However, our results based on dose dependent experiments (Fig 3A and 3B) point at a different scenario where B haplotype carriers have a lower threshold of saturation compared to A haplotype carriers. One mechanistic explanation could be obtained from our calcium analyses. We observed that Store Operated Calcium Entry (SOCE), an early event in the TCR cascade that happens upon calcium store depletion, is diminished in B haplotype carriers in resting cells. The protocol we used to study SOCE is downstream TCR stimulation because it is based on adding cyclopiazonic acid (CPA), an inhibitor of the Sarco Endoplasmic Reticulum Calcium (SERCA) pump that produces the depletion of calcium stores to trigger SOCE. This reduction in calcium entry points at the SOCE machinery as the bottleneck in resting lymphocytes from B haplotype carriers that most likely cause saturation in the transduction of the signal, affecting thus the proliferation rate. Importantly, the fine tuning of the SOCE pathway by channels and regulatory proteins is a hallmark of different lymphocyte subsets and its dysregulation the cause underlying a large number of immune disorders, including autoimmunity [27]. An intriguing idea that emerges from our results is the connection existing between a diminished activation observed in B haplotype, considered the risk haplotype, with the pro-inflammatory phenotype that characterizes asthma pathology. In this respect, it has been reported that Th2 lymphocytes, the T cell subset implicated in asthma disease, show a lower calcium influx compared to other subsets [28,29]. Considering our calcium analysis data, we hypothesize that lymphocytes from haplotype B carriers might be more prone to differentiate to a Th2 subpopulation. Supporting this idea, the risk allele combination of this region has been previously associated with an increased production of Th2 cytokines[11].

At present, we cannot define the key gene of the chromosome region 17q12-q21 involved in the observed effects. Nevertheless, it is likely that *ORMDL3* plays an important role since we have previously shown that the expression of this gene influences the SOCE pathway in a similar manner to what we observe in this study [13]. However, this does not discard the possible contribution of the other genes. In this sense, despite the important role of *IKZF3* in T cell function, it is unlikely to play a role in this process as its expression was not influenced by the haplotype [15,17]. On the contrary *GSDMB* is highly influenced by these *cis* regulatory elements. Its involvement in the mechanism behind the association to inflammatory pathologies deserves further studies given the role of a member of this family of proteins, *GSDMD*, in the inflammasome cascade and pyroptosis [18,19].

The chromosome region 17q12-q21 has been recognized as an important determinant of risk to develop several pro-inflammatory diseases. This study shows a connection between this genetic link and the physiology of the immune system. Our results support the idea that allelic variants in non-coding regions of chromosome 17q12-q21 determine the expression levels of genes nearby and define a different pattern of activation of T lymphocytes. Thus, we observed that individuals carrying the risk haplotype for asthma disease had a lower response to strong stimulation of the TCR complex. We believe that the lower threshold of saturation might influence several essential processes in the T cell physiology, such as T cell maturation, differentiation of different subsets, suppression and, eventually, autoimmunity, increasing thereby the risk to develop inflammatory diseases

## Material and Methods

### Human sample description

Human T cells were isolated from peripheral blood mononuclear cells (PBMC) of healthy volunteers by enhanced human T-Cell immunocolumns (Diagnóstica Longwood, S.L.). The activation of lymphocytes was done using plate-bound anti-CD3 (10μg/ml) kindly provided by Dr. Lopez-Botet and anti-CD28 (BD biosciences).

Genomic DNA was isolated from either T lymphocytes or PBMC and used to sequence two SNPs within the chromosome region 17q12-q21: rs12936231 and rs7216389. Groups from the resulting genotypes were defined as: GG and CC carriers (haplotype A) and CC and TT carriers (haplotype B), for SNPs rs12936231 and rs7216389 respectively. Both groups (haplotype A and B) were composed by healthy individuals. Average age (mean±SD) in each group was: 33±5 years in A carriers group and 30±1 years in B carriers group for RNA experiments; 35±5 years in A carriers group and 26±1 years in B carriers group for calcium experiments; 37±5 years in A carriers group and 30±2 years in B carriers group for proliferation analyses; and 33±5 years in A carriers group and 30±1 years in B carriers group for cytometry. Gender proportion was 57% and 50% females in A and B carriers respectively for RNA experiments, 40% and 40% females in A and B carriers respectively for calcium experiments, 50% and 50% females in A and B carriers respectively for proliferation analyses and 55% and 63% females in A and AB carriers respectively for cytometry experiments. No correlation with age or sex was observed in any parameter evaluated. The study was approved by the respective ethics committees at Hospital Germans Trias I Pujol, and Comite Etico de Investigaciones Clinicas del Instituto Municipal de Asistencia Sanitaria (CEIC-IMAS). All participants provided written informed consent before entering the study.

### Human T cell sample genotyping

Human DNA was extracted using cell lysis buffer containing 100 mM Tris-Cl (pH 7.6), 40 mM EDTA (pH 8.0), 50 mM NaCl, 0.2% SDS and 0.05% Sodium azide. After lysis, a salt solution (1M NaCl final concentration) and ethanol precipitation protocol was used to purify DNA. Genotyping of SNP rs7216389 and SNP rs12936231 was done by amplifying with Biotaq DNA polymerase (Bioline) the surrounding region using primers 5’-GTGCCTGGCATACATTCTAACTGC-3’and 5’-AGCCCTGCCTCCAAAACCTAG-3’; and primers 5’-AGTATGTAGTTGACCTTAGCCTG-3’ and 5’-GGTCCAAGTGCTGAATTCCA-3’, respectively. Cycle sequencing was performed on purified PCR products with an Applied Biosystems BigDye terminator v3.1 sequencing chemistry and run on an ABI 3100 (Applied Biosystems, California, USA) genetic analyzer. The sequences were analyzed with DNAstar Lasergene 11 software.

### Real-time PCR analysis

Extraction of total RNA from human T cells was performed according to manufacturer instructions (Nucleospin RNA II kit, Macherey-Nagel). cDNA was obtained using SuperScript-RT system (Invitrogen) and quantitative PCR was performed on an ABI Prism 7900HT (Applied Biosystems) with SYBR-Green (SYBR-Green Power PCR Master Mix, Applied Biosystems). *ORMDL3* primers were obtained from QuantiTect Primer Assay (Qiagen). Other primers used were the following: *IL-2* 5’-AACTCACCAGGATGCTCACA-3 and 5’-GCACTTCCTCCAGAGGTTTG-3’; *CD25* 5’-CCTGGGACAACCAATGTCA-3’ and 5’-TGGACTTTGCATTTCTGTGG-3’; *CD69* 5’-TCTTTGCATCCGGAGAGTG-3’ and 5’-GCACACAGGACAGGAACTTG-3’; *MLN51* 5’-CAAGGAAGGTCGTGCTGGTT-3 and 5’-ACCAGACCGGCCACCAT-3’; *IKZF3* 5’-TGGAAAATGTGGACAGTGGA-3’ and 5’-CATTTCCCATGGGTTCTGAC-3’; *ZPBP2* 5’- CTGGACAGCTGATGGTGAAA-3’ and 5’-CCCGATAGGCAAAGACCATA-3’; *GSDMB* 5’-ACATGGAGGACCCAGACAAG-3’ and 5’-CACAGAGAATTCGTGCCTCA-3’; *IFNγ* 5’-TGACCAGAGCATCCAAAAGA -3’ and 5’-CTCTTCGACCTCGAAACAGC -3’; *HPRT* 5’-TGACACTGGCAAAACAATGCA-3’ and 5’-GGTCCTTTTCACCAGCAAGCT-3’. *MLN51*, *IKZF3*, *GSDMB* and *ZPBP2* primers were previously described [2,24]. All primers are noted in forward and reverse sequence, respectively. PCR conditions for all cases were 95°C for 5 min; 95°C for 30 s; 60° for 30 s, 72°C for 30 s; and 72°C for 5 min; with 40 cycles of amplification. Normalization of the gene expression analysis was performed to *HPRT* at basal levels (Fig 1A) and to *MLN51* in activation studies (Figs 1B and 1C and 2C–2F).

### Calcium imaging

Cytosolic Ca^2+^ signal was determined in cells loaded with 4,5 μM fura-2 AM (20 min) as previously described [13]. Cytosolic [Ca^2+^] increases are presented as the ratio of emitted fluorescence (510 nm) after excitation at 340 and 380 nm, relative to the ratio measured prior to cell stimulation (fura-2 ratio 340/380). All experiments were carried out at room temperature and cells were bathed in a solution containing (in mM) 140 NaCl, 5 KCl, 1.2 CaCl_2_, 0.5 MgCl_2_, 5 glucose, 10 HEPES (300 mosmol/l, pH 7.4 with Tris). Ca^2+^-free solutions were obtained by replacing CaCl_2_ with an equal amount of MgCl_2_ plus 0.5 mM EGTA. Stores depletion was achieved in human T cells using 30 μM cyclopiazonic acid (CPA).

### Proliferation assays

PBMC were labeled with CFSE dye (Invitrogen) as described [30]. Briefly, cells were incubated with 5μM CFSE (in PBS containing 5% fetal bovine serum (FBS)) at 37°C for 5 min and washed three times with ice-cold PBS-5% FBS. 2x10^5^ CFSE-labeled PBMCs/well of a 96-well plate were cultured in Roswell Park Memorial Institute (RPMI)1640 media supplemented with 10% FBS, 1% penicillin/streptomycin, 1mM Sodium pyruvate and 0.05mM 2-Mercaptoethanol in the presence of decreasing doses of anti-CD3 antibody (ref 555337, BD Pharmigen). PBMC were harvested after 2 and 5 days of culture and stained with anti-CD4-APC and anti-CD8-PE antibodies (eBiosciences) for subsequent analysis by flow cytometry (Fortessa, Becton Dickinson). Percentages of proliferating cells were analyzed by FlowJo software.

### Flow cytometry experiments in peripheral blood mononuclear cells

PBMC from 20 healthy volunteers were isolated from whole peripheral blood using a density gradient (LymphoprepTM, AXIS-SHIELD) centrifugation and frozen until use. Briefly, PBMC were cultured for 3 days in RPMI 1640 media supplemented with 2 mM L-glutamine, 100 U/mL penicillin and 100 μg/mL streptomycin and 20% fetal calf serum (GibcoTM, Life Technologies). For activation, 5 μg/ml PHA (Sigma-Aldrich) and 50 U/ml of recombinant human IL-2 (Roche, Sigma-Aldrich) were added to the media. Viability staining (Live/Dead Fixable Dead Cell Stain kit, Invitrogen) was performed to gate on viable cells and T cell activation was assessed by staining for T cell markers (antibodies: anti-human CD3-APC-H7, CD4-PE-Cy7, and CD8-V500; BD Biosciences) and markers of T cell activation (antibodies: anti-human CD25-APC, HLA-DR-FITC and CD38-PerCP-Cy5.5; BD Biosciences). Cells were collected on an LSR II instrument (Becton Dickinson) and T cell activation markers analysis was performed using the FlowJo software.

## Supporting Information

S1 Fig Expression levels of *MLN51* under lymphocyte activation. *MLN51* amplification expressed in quantification cycles (Cq) obtained by real time PCR reaction of cDNA generated from 100 ng RNA of CD3/CD28-activated T cells from different individuals at 0, 4 and 24 h time points after activation. The number of individuals of haplotype A and haplotype B carriers is indicated in brackets. Data presented as mean ± SD.
(PDF)
