# Supplementary material for: Lymphocyte Activation Dynamics Is Shaped by Hereditary Components at Chromosome Region 17q12-q21
Source: PLoS One. 2016 Nov 11;11(11):e0166414. doi: 10.1371/journal.pone.0166414 (PMC5106028; doi:10.1371/journal.pone.0166414)
Supplement: S1 Fig — MLN51 amplification expressed in quantification cycles (Cq) obtained by real time PCR reaction of cDNA generated from 100 ng RNA of CD3/CD28-activated T cells from different individuals at 0, 4 and 24 h time points after activation. The number of individuals of haplotype A and haplotype B carriers is indicated in brackets. Data presented as mean ± SD. (PDF) [file pone.0166414.s001.pdf]

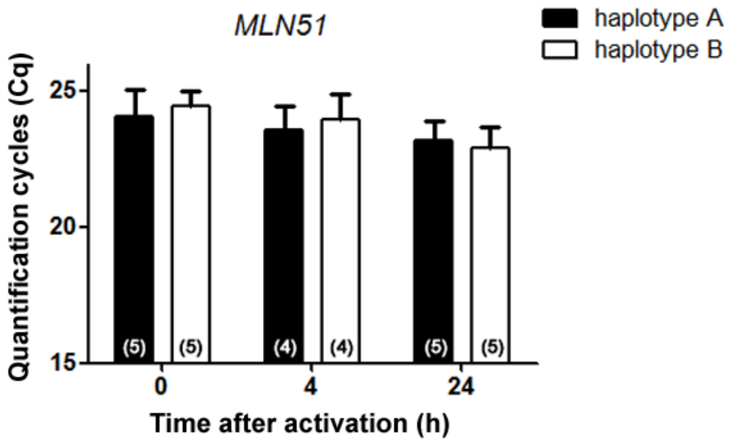

S1 Figure. Expression levels of MLN51 under lymphocyte activation. *MLN51* amplification expressed in quantification cycles (Cq) obtained by real time PCR reaction of cDNA generated from 100 ng RNA of CD3/CD28-activated T cells from different individuals at 0, 4 and 24 h time points after activation. The number of individuals of haplotype A and haplotype B carriers is indicated in brackets. Data presented as mean ± SD.
